# Observation of Hilbert space fragmentation and fractonic excitations in 2D

**DOI:** 10.1038/s41586-024-08188-0

**Published:** 2024-11-13

**Authors:** Daniel Adler, David Wei, Melissa Will, Kritsana Srakaew, Suchita Agrawal, Pascal Weckesser, Roderich Moessner, Frank Pollmann, Immanuel Bloch, Johannes Zeiher

**Affiliations:** 1 Max-Planck-Institut für Quantenoptik, Garching, Germany; 2 Munich Center for Quantum Science and Technology (MCQST), Munich, Germany; 3 Technische Universität München, TUM School of Natural Sciences, Garching, Germany; 4 Max-Planck-Institut für Physik komplexer Systeme, Dresden, Germany; 5 Fakultät für Physik, Ludwig-Maximilians-Universität, Munich, Germany

**Keywords:** Quantum simulation, Statistical physics

## Abstract

The relaxation behaviour of isolated quantum systems taken out of equilibrium is among the most intriguing questions in many-body physics^1^. Quantum systems out of equilibrium typically relax to thermal equilibrium states by scrambling local information and building up entanglement entropy. However, kinetic constraints in the Hamiltonian can lead to a breakdown of this fundamental paradigm owing to a fragmentation of the underlying Hilbert space into dynamically decoupled subsectors in which thermalization can be strongly suppressed^2–5^. Here we experimentally observe Hilbert space fragmentation in a two-dimensional tilted Bose–Hubbard model. Using quantum gas microscopy, we engineer a wide variety of initial states and find a rich set of manifestations of Hilbert space fragmentation involving bulk states, interfaces and defects, that is, two-, one- and zero-dimensional objects. Specifically, uniform initial states with equal particle number and energy differ strikingly in their relaxation dynamics. Inserting controlled defects on top of a global, non-thermalizing chequerboard state, we observe highly anisotropic, subdimensional dynamics, an immediate signature of their fractonic nature^6–9^. An interface between localized and thermalizing states in turn shows dynamics depending on its orientation. Our results mark the observation of Hilbert space fragmentation beyond one dimension, as well as the concomitant direct observation of fractons, and pave the way for in-depth studies of microscopic transport phenomena in constrained systems.

## Abstract (web summary)

Using quantum gas microscopy, Hilbert space fragmentation and fractonic excitations are observed in a two-dimensional tilted Bose–Hubbard model.

## Main

The eigenstate thermalization hypothesis expresses the notion of thermalization in closed quantum systems by stating that eigenstates produce expectation values for local observables that are consistent with those of a thermal ensemble, and thus lose all memory of the initial states during relaxation^10,11^. Recently, several mechanisms have been delineated, where systems defy such thermalizing dynamics and the eigenstate thermalization hypothesis, such as integrability in one-dimensional systems^12,13^, many-body localization in models with quenched disorder^14–16^ or the emergence of many-body scars for specific initial settings^5,17^. Another mechanism is the emergence of kinetic constraints connected with Hilbert space fragmentation (HSF)^2–5,18–21^. In systems showing HSF, a hierarchy of conservation laws exists. First, the full Hilbert space can be divided into (polynomially many) subspaces characterized by global quantum numbers such as particle number or dipole moment. In the corresponding subspaces with constant quantum numbers, local kinetic constraints lead to a further fragmentation of the Hilbert space into exponentially many smaller subsectors, the so-called Krylov subsectors, which cannot be characterized by simple quantum numbers. All states in a single Krylov sector are, by definition, dynamically connected, that is, they can be reached through unitary time evolution with the Hamiltonian^2,3^. One striking consequence of HSF is the possible existence of fragments containing specific states that evade thermalization because of the underlying kinetic constraints and the small size of the associated Krylov sector.

Another particularly interesting consequence of constrained dynamics is the potential emergence of fractons that show restricted mobility^6,7,9^. Fractons can either be immobile under local Hamiltonian dynamics or show subdimensional dynamics, such as propagation in an effectively one-dimensional subspace of two-dimensional space^6–9,22^, as well as anomalous diffusion^22–24^. Previous theoretical studies^2,3,6,7,21,25^ have also related the emergence of fractons to gauge theories associated with local conservation laws and to topological defects in elasticity theory^26^. Relaxation of systems showing fractonic excitations is expected to be strongly impeded, leading to non-ergodic behaviour and strongly temperature-dependent transport dynamics^7,24^. Subdiffusive transport in the tilted Fermi–Hubbard model has recently been observed experimentally^27^. Related theoretical work has connected the emerging subdiffusive hydrodynamic behaviour with the presence of kinetic constraints^22,28^. The kinetic constraints underlying HSF have been experimentally probed directly in one-dimensional tilted Hubbard chains^29,30^. These systems show dipole-moment conservation for strong interactions, as a consequence of the interplay between interaction and tilt energy^2,3,31–33^. Recently, state-specific relaxation behaviour in systems with HSF was also observed in one-dimensional Rydberg arrays kinetically constrained in the facilitation regime^34^ and quantum ladders realized with a superconducting quantum processor^35^. The evasion of thermalization tends to depend, frequently qualitatively, on the underlying dimensionality, particularly for both integrable or disorder-localized systems. Consequently, this naturally motivates the study of the hallmarks of non-ergodicity in higher-dimensional HSF.

**Fig. 1:**
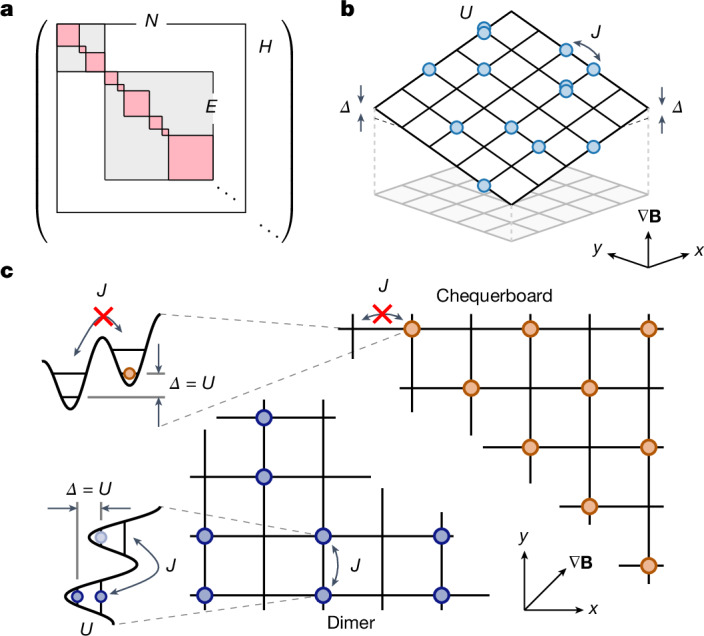
HSF and schematic of the experiment. **a**, The Hilbert space consists of sectors with fixed energy and particle number (conserved quantities), (*E*, *N*) (grey squares). Adding dynamical constraints to the system, these sectors fragment into decoupled Krylov subsectors (pink squares). **b**, Our system is described by a tilted Bose–Hubbard model with a diagonal tilt (realized using a magnetic field** B**) along the *x* + *y* direction tuned to resonance with the interactions, *Δ* = *U*. Tilt and interaction energy are much larger than the tunnel coupling *J*. **c**, Dimer (blue points, bottom left) and chequerboard state (orange points, top right) and first-order processes exemplifying the presence (absence) of density-assisted resonant couplings in the lattice for the dimer (chequerboard) state.

Here we investigate this question for a two-dimensional tilted Bose–Hubbard model, where we study the non-equilibrium dynamics owing to HSF in bulk (2D), interface (1D) and point-like defect (0D) dynamics, and find a rich and interrelated phenomenology. Our experiments leverage the single-site control achievable in a quantum gas microscope to prepare specific initial product states in different Krylov sectors and measure their dynamics after a quantum quench. We find markedly different relaxation dynamics for a chequerboard state and a dimer state (Fig. 1c), which are characterized by the same quantum numbers but are part of different Krylov subsectors. Moreover, we prepare and dynamically track defects on top of the otherwise immobile chequerboard state. Our measurements reveal the fractonic nature of such defects, which manifests itself as strongly constrained, subdimensional motion along a one-dimensional manifold in the two-dimensional system. Finally, we prepare an interface between a chequerboard state and a dimer state and observe strongly asymmetric dynamics across the interface consistent with the fractonic nature of the excitations.

The tilted Bose–Hubbard model has been studied in a number of works theoretically^31,33,36^ and experimentally, focusing on the interesting ground-state phases^32,37^ or emerging long-range tunnelling dynamics^38^. The corresponding Hamiltonian is given by

1 $$\begin{array}{l}\widehat{H}\,=\,-J\sum _{\langle i,j\rangle }{\widehat{a}}_{i}^{\dagger }{\widehat{a}}_{j}+\frac{U}{2}\sum _{i,j}{\widehat{n}}_{i,j}({\widehat{n}}_{i,j}-1)\\ \,\,+\varDelta \sum _{i,j}(i+j){\widehat{n}}_{i,j},\end{array}$$

where ${\widehat{a}}_{i}^{\dagger },{\widehat{a}}_{i}$ are the raising and lowering operators and the sum over $\langle i,j\rangle$ runs over all nearest-neighbour sites while ${\widehat{n}}_{i,j}$ is the number operator on site *i,j*. The tunnel coupling between two sites in the lattice is denoted as *J*, and the interaction energy of two bosons occupying the same site is *U*. Applying a strong tilt along the diagonal of the lattice with *Δ* ≫ *J* introduces dynamical constraints. A particularly interesting regime is reached in the limit *U*/*J* ≫ 1 with resonant tilt *Δ* = *U*. Here both particle number *N* and the sum of tilt and interaction energy, *E* = ∑_*i*_*Δ*_*i*_ + *U*_*i*_, with *Δ*_*i*_ and *U*_*i*_ the local values of *Δ* and *U* on site *i*, are approximately conserved globally such that sectors with fixed quantum numbers (*E*, *N*) emerge^8^ (Fig. 1a). In addition, atoms can only couple resonantly to already occupied sites and are thus subject to strong dynamical constraints. Retaining only terms up to and including second order in *J*/*U*, these constraints have been recently shown to result in HSF^8^. In particular, HSF can be observed in first order in this model, which experimentally allows access to longer timescales compared with other models showing HSF based on second-order processes^2,3^. Two states of a single sector with fixed (*E*, *N*) that are expected to show markedly different thermalization behaviour are the chequerboard state and the dimer state shown in Fig. 1c. In the chequerboard state, isolated atoms are not coupled to neighbouring sites, and they are expected to remain frozen and retain memory of the initial density pattern. This contrasts with the dimer state, which is characterized by neighbouring pairs and thus features resonances *Δ* = *U* that can facilitate dynamics and thus lead to a relaxation of the initial density pattern.

**Extended Data Fig. 1:**
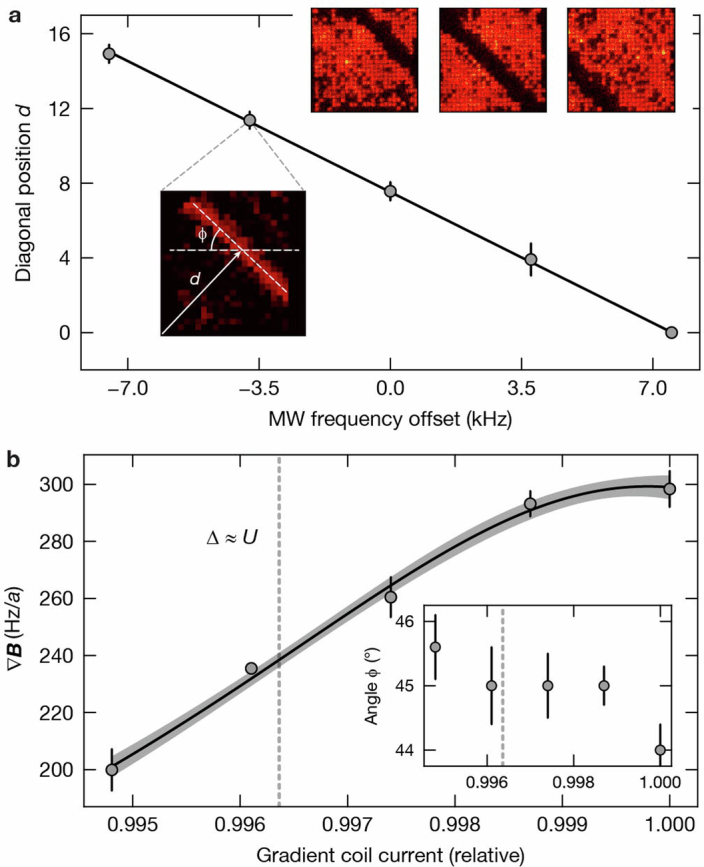
Magnetic field gradient calibration. **a** Fitted center positions of the spatial profiles; gradient calibration (strength and orientation). Insets show exemplary single shots of the microwave (MW) resonances for different centre frequencies. These shots are averaged and fitted with a 2D Gaussian to extract the gradient strength and orientation. **b** Gradient strength from the analysis in Extended Data Fig. 1a at different gradient coil settings. The magnetic field is interpolated using Eq. (3). The inset shows that the angle does not change significantly even when tuning the gradient. The vertical, dashed lines indicate the operating point chosen based on the measurement in Extended Data Fig. 2.

**Extended Data Fig. 2:**
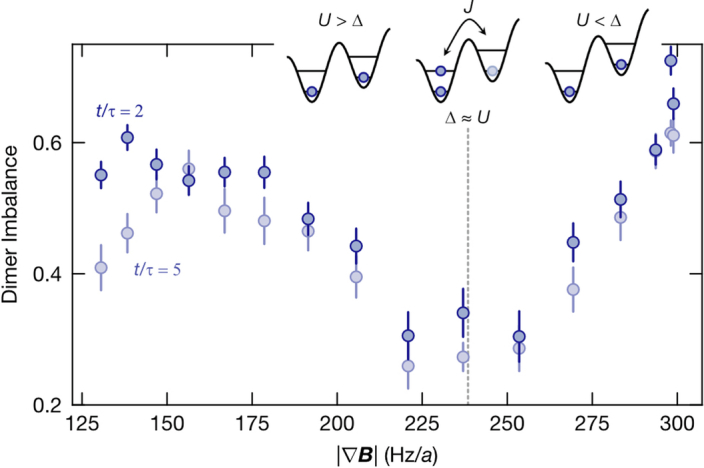
Dimer imbalance for different gradient strengths. Imbalance of the dimer at *t*/*τ* = 2 (dark blue circles) and *t*/*τ* = 5 (light blue circles) for different gradient strengths. The vertical line indicates the operating point chosen for all other measurements. Insets illustrate our expectations about the functional shape of the imbalance (see text) based on the possible processes. Error bars denote the standard error of the mean (s.e.m.).

We start our experiments by preparing a near-unity-filled Mott insulator of about 200 bosonic ^87^Rb atoms in the $| F=1,{m}_{F}=-\,1\rangle$ ground state (where *F* is the total angular momentum and* m*_*F*_ is the Zeeman sublevel) in a single slice of a vertical optical lattice. In the two-dimensional plane, we set the Hubbard parameters by controlling the depth of a two-dimensional folded horizontal lattice^39^. We use a digital micromirror device to reduce the harmonic confinement induced by the optical lattice beams and realize approximately homogeneous trapping conditions^40^. We then exploit single-site addressing^41,42^ to prepare different initial states in sectors with fixed energy and particle number (*E*, *N*). Next, we adiabatically ramp up a potential gradient using a magnetic field to the resonance condition *Δ* ≈ *U* and then quench the lattice depth to *U*/*J* ≫ 1, initiating dynamics (Methods and Extended Data Figs. 1 and 2). After a variable evolution time, we rapidly ramp up the lattice to freeze the dynamics and then record a fluorescence image of the parity-projected occupation per lattice site^43^.

**Fig. 2:**
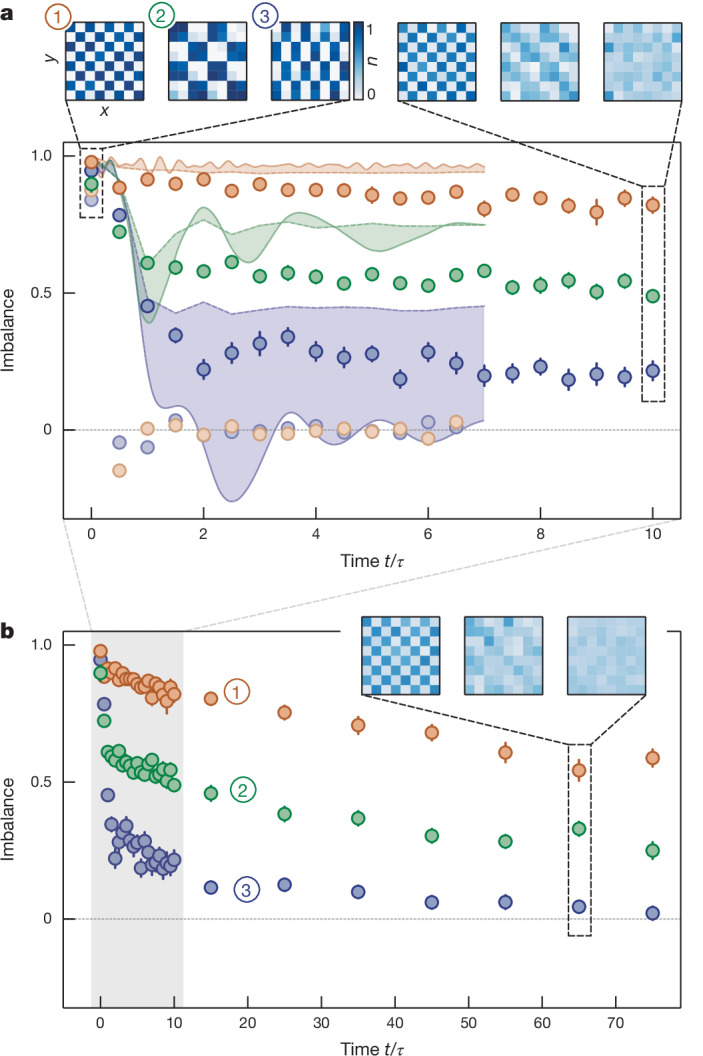
Relaxation of the imbalance for different initial states. **a**, Short-time evolution of the imbalance. Imbalance of different initial states as a function of evolution time in units of the hopping timescale *τ* for the chequerboard (orange), squares (green) and dimer (blue) initial states with (circles) and without (desaturated circles) applied tilt. The imbalance in the case without tilt quickly decays to zero regardless of the initial state, whereas the decay strongly depends on the initial state once the tilt is applied, a clear signature of HSF. Insets: average densities *n* corresponding to the respective states at the indicated times in the 8 × 8 sites region of interest. The shaded, coloured areas denote the areas between theoretical calculations under imperfect (dashed lines) and perfect (solid lines) conditions. Theoretical data were obtained using TeNPy^46,47^ (for details, see Methods). **b**, Imbalance for longer evolution times. The grey shaded area highlights the data points shown in **a**. All error bars denote the standard error of the mean.

In a first set of measurements, we aimed to directly show the emergence of HSF through the vastly different dynamics of different initial states in our model^8^. We prepare the chequerboard state, the dimer state and also the ‘squares’ state, a chequerboard-like arrangement where four atoms and four empty sites, respectively, form the building blocks of a larger chequerboard-like structure. For perfect initial-state preparation, all of these states have the same energy and particle number. The chequerboard state is part of a small fragment, dynamically disconnected from all other states, and thus frozen, whereas the dimer state is part of the largest fragment of the Hilbert space. The squares state is expected to lie in-between, that is, it is part of a larger but not the largest fragment. To probe the relaxation behaviour for each pattern, we evaluate the imbalance defined as

2 $${\mathcal{I}}=\frac{{N}_{{\rm{o}}}-{N}_{{\rm{u}}}}{{N}_{{\rm{o}}}+{N}_{{\rm{u}}}}$$

where *N*_o_ and *N*_u_ are the parity-projected, detected number of atoms on initially occupied and unoccupied sites, respectively. The imbalance captures the degree to which the system retains a memory of the initially prepared pattern. Tracking the evolution of the states in Fig. 2, we find that for the chequerboard, the imbalance is finite and large even for the longest evolution times up to *t*/*τ* = 80, where *τ* = *ħ*/*J* denotes the timescale associated with tunnelling in our experiment (where ℏ is Planck’s constant divided by 2π). We attribute the initial small decay of the chequerboard imbalance within a few *τ* to imperfect preparation of the initial state and higher-order processes^8^. By contrast, the dimer state initially decays much faster to a strikingly lower imbalance, which then only slowly decays towards zero for the longest evolution times. The imbalance of the squares pattern is found to lie approximately between the two extremal cases. Interestingly, analysing the density at the largest evolution times, we observe that the residual imbalance for the squares pattern is due to a larger-scale structure in the density formed in particular by sites that are inaccessible for the atoms owing to the presence of kinetic constraints. For details about the presented numerical simulations, see Methods and Extended Data Fig. 7.

**Fig. 3:**
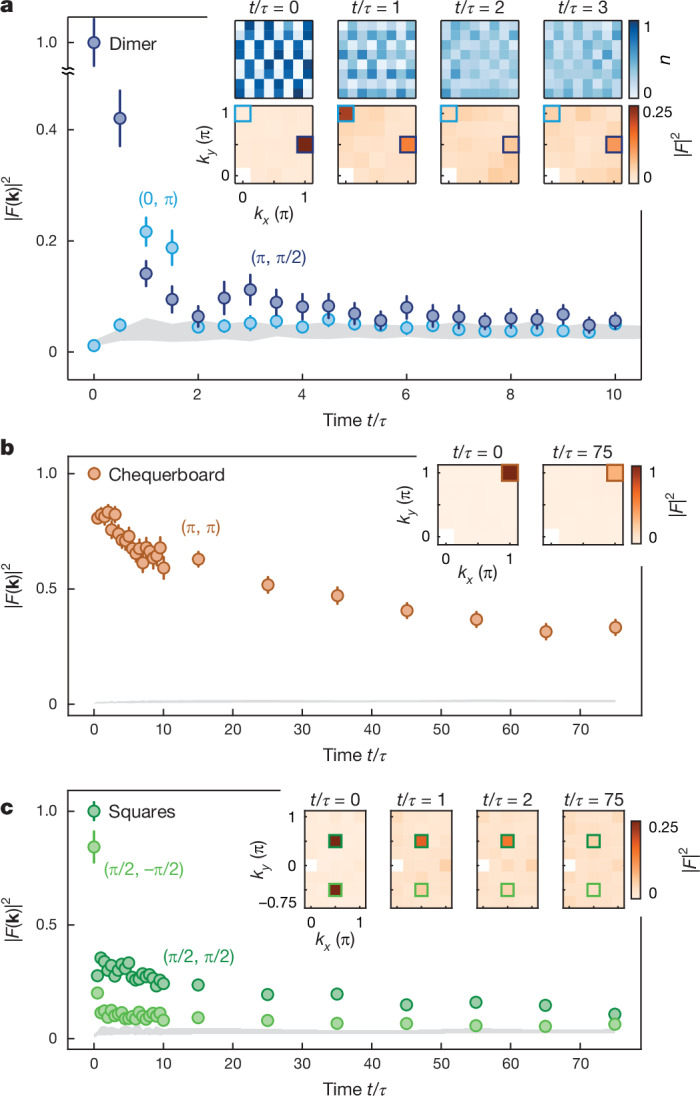
Microscopic study of relaxation. **a**, Fourier analysis of the average densities for the dimer state. The (π, π/2) Fourier component corresponding to the dimer state (dark blue) shows a fast decay, whereas the (0, π) Fourier component for the CDW along the vertical direction (light blue) increases before decreasing again, corresponding to the first hopping processes. **b**, Fourier analysis for the chequerboard state. The (π, π) component decays only slightly and remains the dominant component. **c**, Fourier analysis for the squares state. Both the (π/2, −π/2) and the (π/2, π/2) components decay quickly. The (π/2, −π/2) component, which describes decay in the direction of the equipotential lines, decays to a lower value and quickly becomes indistinguishable from the background, whereas the (π/2, π/2) component is above the background even at late times. For all initial states, all other components fall in-between the grey shaded areas describing the homogeneous background. Insets: the discrete two-dimensional Fourier transforms *F*(**k**) (orange colourmap) with Fourier modes **k** of the average densities (blue colourmap) for selected times. The coloured rectangles highlight the Fourier components shown in the plots. Error bars denote the standard error of the mean.

**Extended Data Fig. 3:**
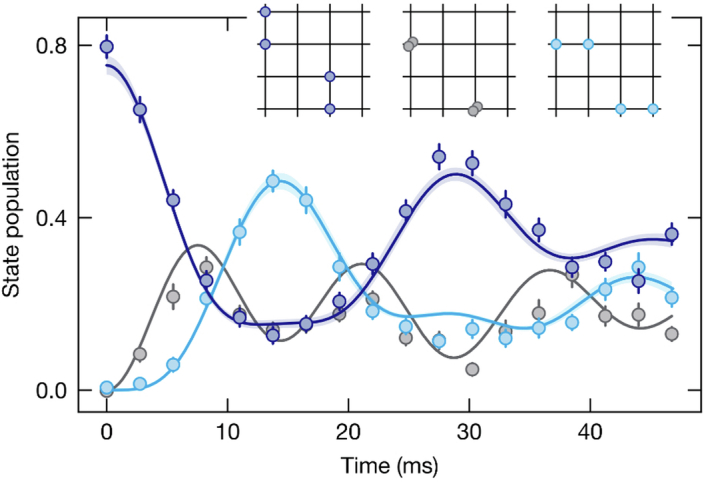
Dynamics of isolated dimers. Preparing an initial state of dimers with additional free sites, we can study the dynamics of isolated dimer pairs and clearly resolve the oscillatory behavior between the initially prepared vertical (dark blue) and the flipped, horizontal (light blue) dimers. The blue solid lines are fits to the data according to a three state model. Additionally, we can also resolve the occupation of the (grey) doublon state. The grey solid line is the expected doublon occupation for the ideal fit parameters and serves as a guide to the eye. All error bars denote the s.e.m.

The relaxation of the dimer state occurs microscopically through a resonant three-site subsystem that is initially connected via first-order tunnelling, which effectively allows the dimers to flip their orientation. These processes are clearly visible in the time evolution of the density (Fig. 3a), where the initial dimer pattern evolves into a stripe-like pattern resembling a charge density wave (CDW) before evolving back into the dimer pattern. This characteristic relaxation behaviour also becomes apparent in a Fourier analysis of the density. Following a quick initial decay of the initial Fourier component (π, π/2) characterizing the dimer state, we observe the growth of the (0, π) component corresponding to a CDW along the *y* direction. Subsequently, we also observe a small revival of the initial dimer pattern during the relaxation dynamics, both in density and Fourier component (Fig. 3a and Extended Data Fig. 3). In stark contrast, for the chequerboard state, the (π, π) component remains the dominant Fourier component at all times and shows a fast initial decay followed by a slow decrease at long times. For the squares initial state, two Fourier components with orthogonal orientation are relevant. First, a fast relaxation occurs within the squares, whereas the coupling between different squares leads to a further slow decay of the (π/2, π/2) and (π/2, −π/2) components (as shown in Fig. 3c), consistent with the slow relaxation of the imbalance. Here the (π/2, −π/2) component shows a faster decay compared with the (π/2, π/2) component and becomes consistent with the ‘background’ of all other components at late times. By contrast, the (π/2, π/2) component is above the background level at all times. This is owing to the faster decay of the initial state along the direction of the equipotential lines, which corresponds to the (π/2, −π/2) component. Orthogonal to this direction, as described by the (π/2, π/2) component, the kinetic constraints inhibit this decay, as is also visible in the inset in Fig. 2b.

**Fig. 4:**
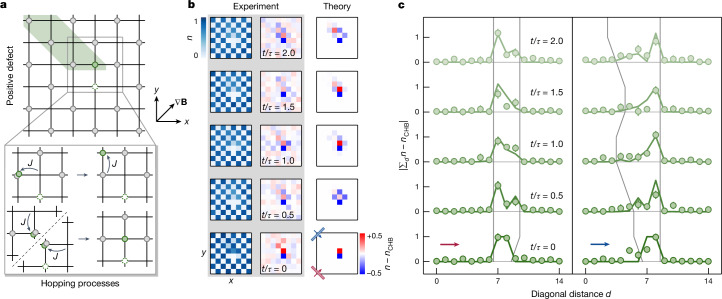
Dynamics of fractonic excitations (positive defect). **a**, Schematic of the spreading of the defect atom (filled green circle) to first order, which can move by forming doublons (dark-green shaded area). On short timescales (to first order), the associated defect hole (dashed circle) is stuck. **b**, Anisotropic spreading of the defect. The spreading of the defect can be observed in the occupations (owing to parity projection) and more clearly in the difference plots when subtracting the time-evolved reference chequerboard (CHB) background. Theory calculations are shown for comparison (Methods). The motion of the defect atom is restricted to a narrow stripe, along which it can move in only one direction. **c**, Diagonal sums over the reference-subtracted occupations. Orthogonal to the equipotential lines (left), the defect atom cannot spread, whereas parallel to the equipotential lines (right), it shows asymmetric propagation on the subdimensional manifold. Grey lines are guides to the eye, based on the points (sampled every 0.25 *t*/*τ*) where the theory calculations (green solid lines) exceed a value larger than 0.05. The coloured arrows show the direction of the summation of the diagonals, as indicated by the insets in the bottom, rightmost plot in **b**. Error bars denote the standard error of the mean.

**Extended Data Fig. 4:**
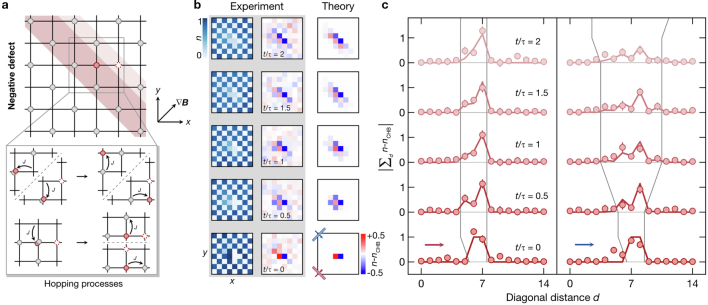
Dynamics of fractonic excitations (negative defect). **a** Schematic of the spreading of the defect atom (filled red circle) to first order, which can move by forming doublons (dark red shaded area). The defect hole (dashed circle) can also move, facilitated by the defect atom (light red shaded area). **b** Anisotropic spreading of the defect analyzed in analogy to Fig. 4. **c** Sums over the reference-subtracted occupations, analyzed and compared to theory (red solid lines) in analogy to Fig. 4. Compared with the positive defect, the negative defect exhibits symmetric propagation on the sub-dimensional manifold. All error bars denote the s.e.m.

**Extended Data Fig. 5:**
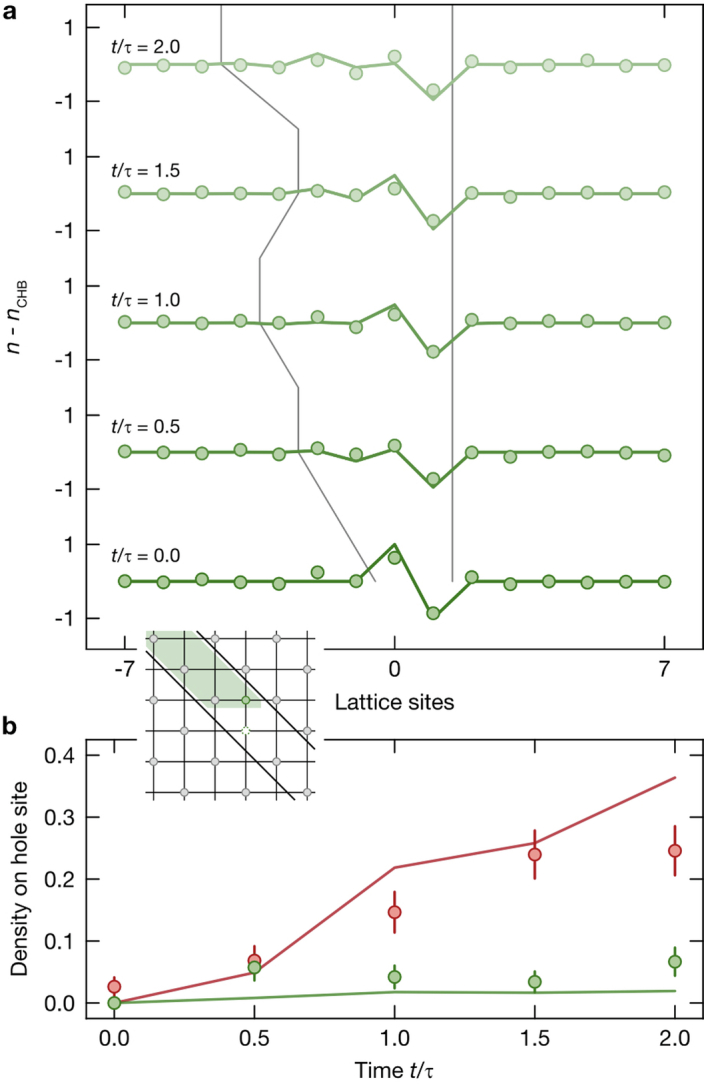
Additional defect data. **a** Positive defect on equipotential surface. Spreading of the defect on the zigzag-shaped equipotential line bounded by the black lines shown in the inset. The defect atom can move, but only in one direction, whereas the hole remains immobile. Grey lines are guides to the eye, based on the points (sampled every 0.25 *t*/*τ*) where the absolute value of the theory calculation (green solid lines) is larger than 0.03. Error bars are on the order of the marker sizes. **b** Density on the site initially occupied by the defect hole. For the negative defect (red), the hole can move in first order, whereas the hole of the positive defect (green) is stuck. Solid lines are theory calculations. Error bars denote the s.e.m.

**Extended Data Fig. 6:**
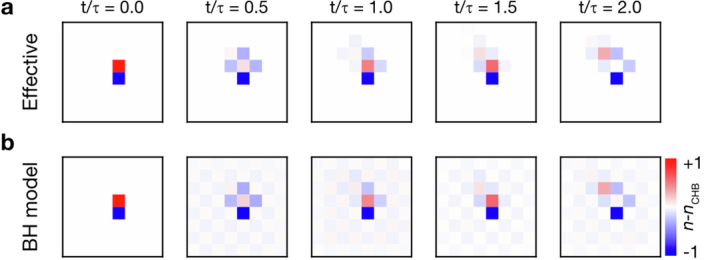
Dynamics of the positive defect. All figures show the parity projected number of particles over time subtracted by a perfect (at *t*/*τ* = 0) chequerboard state. **a** Time evolution with the effective Hamiltonian derived in ref. ^8^ using ED. The background chequerboard pattern is completely frozen, and only the defect is mobile along the diagonal. **b** MPO time-evolution with the full Bose-Hubbard model shows great agreement with the effective time-evolution. In contrast to the effective model, the background chequerboard is not completely frozen. Parameters used: d*t* = 0.0005, *χ* = 256, *N*_max_ = 2.

After establishing the strong dependence of the observed dynamics on the initially prepared state, we aimed to study the dynamics of excitations on top of the fragmented states. Owing to the kinetically constrained dynamics, defects prepared on top of the chequerboard state are expected to show fractonic behaviour^8^. To prepare ‘positive’ (‘negative’) defects, we displace one atom in the chequerboard state by one site such that its energy with respect to the tilt is increased (decreased). As shown in Fig. 4a for the positive defect, the displacement leads to the emergence of new resonant processes, rendering the defect mobile with a subsequent dynamical evolution. We make two striking observations when tracking the dynamics of positive and negative defects following a quench to finite tunnelling. First, both types of defect are confined to movements in a one-dimensional subspace along the equipotential lines on the lattice grid, as shown in Fig. 4c, left (see Methods and Extended Data Fig. 4 for the negative defect). Second, within this one-dimensional subspace, the positive defect propagates asymmetrically to only one side, which can be understood from the presence of the hole associated with the defect, which results in a blocked site in the direct vicinity of the prepared defect (Fig. 4c, right). Only for the positive defect, this hole is immobile to first order and, for the times studied here, remains at the site where it was originally created (Methods and Extended Data Fig. 5). The combination of blocked site and immobile hole observed in our experiment thus directly explains the asymmetric expansion. We can observe the (asymmetric) propagation in the one-dimensional subspace also directly in the average occupation as a reduction of the chequerboard contrast in the direction where the defect can move (Fig. 4b; see Methods and Extended Data Fig. 4 for the negative defect). The subdimensional propagation for both the positive and the negative defects and the unidirectional motion of the positive defect are strong indications that the prepared defects indeed show the expected fractonic properties. For details about the presented numerical simulations, see Methods and Extended Data Fig. 6.

**Fig. 5:**
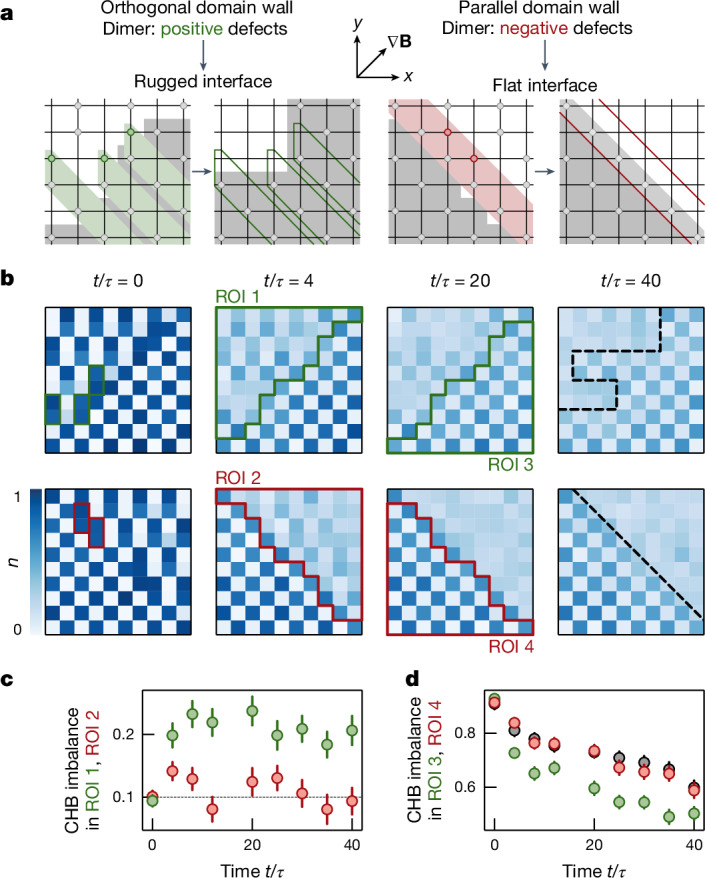
Domain wall dynamics between the chequerboard and dimer states. **a**, Depending on the interface orientation relative to the tilt (orthogonal, left; parallel, right), the dimers act as positive (green circles) or negative (red circles) defects in the chequerboard (grey area). At the interface, the upper atom in the dimers can propagate, whereas the lower atom stays at its original site. **b**, Mean density as a function of evolution time for the central 10 × 10 sites. We observe the emergence of a larger chequerboard area for the orthogonal orientation (top row), indicated by the black dashed line at *t*/*τ* = 40. For the parallel orientation, the chequerboard remains intact. **c**, Evaluating the data in the green and red triangular regions of interest (chosen such that there is an even, identical number of sites in both halves of the system; Methods) illustrated in **b**, we find that the overlap with the chequerboard (CHB) remains constant for the parallel orientation (red) whereas we observe an increase for the orthogonal orientation (green). **d**, For the orthogonal interface orientation (green), the imbalance in the chequerboard half of the system decays. For the parallel orientation (red), the imbalance stays consistent with the reference measurement of a pure chequerboard (grey). All error bars denote the standard error of the mean. ROI, region of interest.

In a final set of measurements, we studied the relaxation of an interface between the mobile dimer state and the immobile chequerboard state. Such a measurement directly probes the impact of kinetic constraints for the underlying defects on transport characteristics in systems showing HSF. Interestingly, we observe strikingly different dynamics depending on the alignment of the interface along or orthogonal to the equipotential surfaces. If the interface is aligned with the equipotential surface, the chequerboard state remains stable, whereas the dimer rapidly decays. Importantly, the interface stays intact, which indicates that—consistent with the subdimensional character of the defects—no transport occurs across the boundary. Conversely, if the interface is oriented orthogonal to the equipotential lines, a chequerboard structure starts building up even on the sites initially prepared with a dimer pattern. These observations can be directly explained by the type and location of defects initially injected into the system, in combination with the strongly asymmetric fractonic character of single defects (Fig. 5a). In the vicinity of the interface, the mobile atom in each initially prepared dimer will propagate into the chequerboard region. The remaining atoms in these dimers form a chequerboard pattern, effectively increasing the overall area of the then immobile chequerboard. This is evidenced by the increase of the chequerboard imbalance evaluated in the half of the system initially prepared in the dimer state (Fig. 5c). Simultaneously, as shown in Fig. 5d, the chequerboard imbalance within the other half of the system drops significantly lower than for an independent reference chequerboard measurement without an interface or for the interface orientation parallel to the equipotential lines, owing to the influence of the mobile defects.

In summary, we have demonstrated the emergence of HSF in a two-dimensional tilted Bose–Hubbard model as a consequence of strong kinetic constraints. Our measurements provide a comprehensive study of HSF in two-dimensional systems through their thermalization properties, which differ strikingly for different initial states. Our results immediately raise follow-up questions, such as whether the tilted Bose–Hubbard model is weakly or strongly fragmented. Such a study would require measurements for much larger system sizes and a finite-size scaling analysis of the final imbalance, or the preparation of a number of other initial states from one (*E*, *N*) subsector. It would also be interesting to study the predicted diffusive and subdiffusive behaviour of negative and positive defects^8^, which would be possible in larger systems with longer coherence times. Furthermore, the detailed understanding of the complex dynamics emerging at the interface between states from different fragments remains open and is left for further work, stressing that numerical simulations are limited to evolution times much smaller than those accessible in our experiment. In future work, it may also be interesting to study HSF in open systems experimentally by adding controlled dissipation^44^, or explore quantum HSF, where the Hilbert space fragments are characterized by entangled substates^45^.

## Methods

### Experimental details

Here we briefly describe the initial-state preparation common to all measurements. Experiments were performed in a single plane of a vertical one-dimensional optical lattice. For the in-plane lattice, we used the folded lattice described in ref. ^39^. As the in-plane lattice is subject to disorder and harmonic confinement, we used a digital micromirror device to shape the horizontal on-site potential, allowing us to achieve approximately homogeneous trapping depths and tunnelling energies throughout the system. Using a second digital micromirror device, we additionally projected a tapered, rectangular box in the centre of this corrected system, to achieve reliable loading and high filling in a central area of about 15 × 15 lattice sites.

Starting from these Mott insulators, to prepare the initial states of interest, we then performed local addressing over the entire area^41,42^, whereas the data analysis was performed in a smaller region of interest (ROI) of either 8 × 8 or 10 × 10 lattice sites at the centre of the system. In addition, working with larger systems than the size of the ROI minimizes the influence of finite-size and boundary effects. With this preparation sequence, we achieved a filling of 0.88(2) per site on the addressed sites and a filling of 0.04(2) on the non-addressed sites in the ROI. These values were averaged over all datasets and initial configurations.

### Magnetic-field gradient calibration

The potential tilt in our experiments was realized by global magnetic fields, which allowed us to induce the most homogeneous gradients. We calibrated the magnitude and the orientation of the magnetic gradient using spatially resolved microwave spectroscopy on the magnetic-field-sensitive transition between the $| F=1,{m}_{F}=-\,1\rangle$ and the $| F=2,{m}_{F}=-\,2\rangle$ hyperfine ground states.

To this end, we prepared a large Mott insulator with all atoms in the $| F=1,{m}_{F}=-\,1| \rangle$ state. We then adiabatically ramped up the magnetic field to its target configuration and performed narrow microwave sweeps at variable centre frequencies. As a consequence, atoms were addressed resonantly within a narrow stripe subjected to the same magnetic-field strength and flipped into the $| F=2,{m}_{F}=-\,2\rangle$ state, which were then removed before imaging. We fitted a two-dimensional Gaussian to these stripes of missing atoms, which allowed us to map the field strength and gradient orientation versus their position (Extended Data Fig. 1a).

To be able to continuously vary the applied gradient strength, we used a combination of coils: a single vertical gradient coil and a pair of vertical offset coils in Helmholtz configuration with reversed field polarity to realize a quadrupole field near the plane of the atoms. For the initial calibration, we worked with a fixed gradient coil setpoint and tuned the vertical offset and additional in-plane offset fields such that the magnetic zero point was at the location of the atoms; subsequently, we shifted the zero point by a fixed amount using the in-plane offset fields, resulting in an in-plane gradient at the correct angle. We then proceeded to calibrate the gradient strength for various gradient coil setpoints as described above; for technical reasons, we tuned the gradient coil instead of the offset coils. We interpolated between the calibrated values by fitting them with the function

3 $$g(\Delta B)=\frac{{g}_{0}^{2}r}{\sqrt{{g}_{0}^{2}{r}^{2}+{(\Delta B+{B}_{0})}^{2}}},$$

where *r* is the displacement of the magnetic field zero to the atoms, *g*_0_ is the maximal gradient strength, *B*_0_ describes background fields and Δ*B* is the change of the setpoint of the gradient coil. As we changed Δ*B* by only a few per cent, we can assume *g*_0_(*B*) = constant, which is also supported by the fact that the fit function describes the data well, as shown in Extended Data Fig. 1b.

On the basis of this curve, we can then rescale the *x* axis in Extended Data Fig. 2 and obtain an absolute value for the gradient strength.

### Hubbard parameters

To extract the Hubbard parameters of our folded optical lattice^39^, we made use of two methods. First, we performed amplitude modulation spectroscopy to calibrate the lattice depth. The results were then compared with a band-structure calculation to obtain the values for the on-site interaction *U* and the tunnelling energy *J*. Here we found *U*/*J* = 21(2) with *U* = *h* × 275(5) Hz and *J* = *h* × 13(1) Hz. The error bars arise from the uncertainty of the lattice-depth calibration itself as well as the slightly anisotropic hopping along the two lattice axes^39^. Second, we can independently calibrate the Hubbard parameters using the quench dynamics of isolated dimers (see Extended Data Fig. 3 and below). As a result, we extracted *τ* = *ħ*/*J* = 10.0(3) ms, equivalent to *J* = *h* × 16.0(5) Hz. Comparing again with our band-structure calculation, this corresponds to *U*/*J* = 17(1) with *U* = *h* × 260(5) Hz. We attribute the deviations between these two calibrations to day-to-day drifts of the lattice beam alignment over the entire data-taking period.

For data evaluation, we used *τ* = 11 ms for all datasets, motivated by the long data-taking period of several days for a given dataset. Theory calculations (see below) were performed for *U*/*J* = 18, which was chosen as an intermediate value between the two calibrations.

### Tuning the gradient to resonance

For the presented studies, it is important that the applied gradient matches the on-site interaction, that is, *Δ* = *U*. We benchmarked the resonance location by measuring the dimer imbalance as a function of the gradient strength for various tunnelling times, as illustrated in Extended Data Fig. 2. Here we expected the strongest decay of the dimer imbalance, as defined in the main text, when the resonance condition is fulfilled. For smaller gradients, we expected a slower drop in imbalance, whereas for much stronger gradients, we expected all processes to be off-resonant and no dynamics to occur at all, leading to high imbalance even at later times.

Our experimental results match the described expectation qualitatively. To confirm that we were not accidentally probing at a time where the imbalance shows any *Δ*-dependent oscillations, we probed for multiple fixed evolution times (up to *t*/*τ* = 40), observing consistent behaviour for all of the chosen evolution times. The resonance width is inherently limited by the finite tunnelling bandwidth and residual potential disorder. Our chosen operation point was located at the centre of the resonance and showed the strongest decay, as marked by the vertical dashed line in Extended Data Fig. 2. On the basis of our gradient calibration presented above and in Extended Data Fig. 1b, this point corresponds to a value of *Δ* = *h* × 238(3) Hz.

Comparing with our independent band-structure calculation, we found a qualitative agreement within 15% to the value of *U* for both calibration methods of the Hubbard parameters described above. In particular, *U* changes only very slowly with the lattice depth and varies by less than *J* for our calibrations. As such, this gradient setpoint remains valid throughout all measurements.

### Data analysis

All data, unless specified differently, were analysed as explained in the following: we calculated the quantity of interest (imbalance, Fourier components, diagonal sums) on the individual experimental shots, then averaged over these results to obtain the data shown in the figures. For the reference-subtracted defect occupations (Fig. 4b, middle, and Extended Data Fig. 4b), we subtracted the densities averaged over all shots. To calculate the imbalances in Fig. 5c,d, we chose the boundary of the respective ROIs such that atoms close to the interface boundary that could be part of either the chequerboard or the dimer were counted as belonging to the dimer part of the system. As such, we obtained the same number of atoms for both halves of the system and the imbalance can, in principle, reach its typical limits of ±1; this explains the perhaps unintuitive shape of the ROIs shown in Fig. 5b.

### Fourier analysis

To analyse the Fourier components of the average densities, we calculated the discrete Fourier transform according to

4 $$\begin{array}{l}F({\bf{k}})\,=\,\mathop{\sum }\limits_{n=0}^{N-1}\mathop{\sum }\limits_{m=0}^{M-1}{a}_{n,m}\exp \,\left(-2{\rm{\pi }}i(\frac{nj}{N}+\frac{ml}{M})\right)\\ \,\,=\mathop{\sum }\limits_{n=0}^{N-1}\mathop{\sum }\limits_{m=0}^{M-1}{a}_{n,m}\exp (\,-\,{\rm{i}}{k}_{x}n-{\rm{i}}{k}_{y}m)\end{array}$$

with *a*_*n*,*m*_ the average densities at site *n*, *m* for an ROI of size *N* × *M* and ${k}_{x}=\frac{2{\rm{\pi }}j}{N},{k}_{y}=\frac{2{\rm{\pi }}l}{M}$. Here the index *j* runs from $-\left(\frac{N-1}{2}\right),\ldots ,0,\ldots ,\frac{N-1}{2}$ for odd *N* and $\lceil \frac{N-1}{2}\rceil ,\ldots ,0,\ldots ,\frac{N}{2}$ for even *N* and analogously for *l*. The value at (*k*_*x*_, *k*_*y*_) = (0, 0) is just the sum of the signal; it contains no additional relevant information and is thus neglected (white rectangles in the insets of Fig. 3).

It is noted that the discrete Fourier transform obeys point reflection symmetry, that is, *F*(**k**) = *F*(−**k**). Therefore, in the main text, we plot only the parts of the momentum space (*k*_*x*_, *k*_*y*_) that contain non-redundant information.

### Isolated dimer dynamics

To further understand and investigate the decay of the dimer pattern on a microscopic level, we prepared isolated dimers and tracked their evolution after a sudden quench. We isolated the dimers by adding empty columns between the atom pairs, as illustrated in the inset of Extended Data Fig. 3. For this configuration, the dimers were, including only first-order processes, completely decoupled from one another, allowing us to study the formation of the horizontally oriented dimers described in Fig. 3a. The change in orientation can be understood intuitively. Starting from a dimer, the upper atom can tunnel onto the neighbouring site by forming a doublon, as illustrated in the middle inset of Extended Data Fig. 3. From there, the atoms can either rearrange into the original dimer or into the flipped dimer, which is energetically degenerate to the original dimer configuration.

Extended Data Fig. 3 shows the time evolution of the isolated dimers. Here we plot the populations of the three possible states: the vertical dimer, the doublon and the horizontal dimer. Although the dimer states can be detected unambiguously, we assigned the doublon if all three sites were empty. To correct, on average, for cases where no atoms were initially prepared, we subtracted the value obtained analogously from a reference measurement tracking the initial-state preparation. We observed a clear oscillation between the two cases of vertically and horizontally oriented dimers, which quickly dephases owing to residual potential disorder. We compared the measured data with a numerical simulation of a three-state model given by

5 $$\widehat{H}=\left(\begin{array}{ccc}{\delta }_{i} & \sqrt{2}J & 0\\ \sqrt{2}J & U-\varDelta +{\delta }_{j} & \sqrt{2}J\\ 0 & \sqrt{2}J & {\delta }_{k}\end{array}\right)$$

where *δ*_*i*_, *δ*_*j*_, *δ*_*k*_ describe the disorder strength between adjacent sites. For the calculation, we sampled *δ*_*i*_, *δ*_*j*_, *δ*_*k*_ from a normal distribution around zero and averaged over *N* = 100 such realizations. The additional factor of $\sqrt{2}$ for the hopping has to be taken into account owing to the bosonic enhancement characteristic for indistinguishable bosons. We then fitted the calculations to the measured occupation of the vertical and horizontal dimers to generate the solid lines in Fig. 3b. Here we allowed for the disorder strength, the difference *U* − *Δ*, an overall amplitude (which respects normalization) as well as the timescale as free fit parameters. The initial time offset was kept fixed at zero. It is noted that the doublon occupation was not included in the fits, instead the solid line in Extended Data Fig. 3 is given by the model expectation using the fit values obtained from fitting the two other curves. We observed good agreement between the doublon occupation as obtained from our measured data and the numerical model using the fit parameters for the two other curves, validating our method of extracting the doublon occupation.

From the fit, we extracted the standard deviation of the disorder distribution *σ* = 1.2(1) × *J*, a deviation from resonance of *U* − *Δ* = 0.0(3) × *J* and a timescale of *τ* = 10.0(3) ms. The latter can serve as a secondary way to calibrate the Hubbard parameters of our system (see above).

### Negative defect and additional analysis

Here we present our measurements on the negative defect and describe the data presented in Fig. 4 and Extended Data Fig. 4 in more detail. We also present an alternative way of evaluating the data for the positive defect and directly compare the spreading of the defect holes for both the negative and the positive defects.

The spreading of the defects can be observed directly in the average occupations (Fig. 4b and Extended Data Fig. 4b, leftmost column), through a reduced contrast of the (background) chequerboard on sites accessible to the defect atoms. This is owing to the following processes. First, the defect atoms can move to initially empty sites of the chequerboard, thereby increasing the average density on these sites. The defect atoms can also move onto initially occupied sites of the chequerboard, where we then observe a reduced average density owing to parity projection. Finally, nearby atoms from the background chequerboard can become mobile owing to the presence of the defect and move onto the site occupied by the defect atom, thus reducing the average density on their original sites as well as on the site of the defect atom owing to parity projection. For the negative defect in particular, the motion of the hole can be observed by an increase of the average occupation on its initial site (see also Extended Data Fig. 5b in the following), and a simultaneous decrease of the average occupation on the neighbouring sites on its equipotential line. By contrast, the hole site for the positive defect remains unoccupied. These effects are highlighted by subtracting the occupation of a chequerboard state without deterministically created defects. Initially empty sites accessible to the defect atoms will feature a positive, reference-subtracted value, whereas initially occupied sites accessible to the defect atoms will show negative values. The latter, as explained above, is due to either parity projection, atoms becoming mobile owing to the defect or, for the case of the negative defect, also the spreading of the defect hole. When comparing with theory, we observed good agreement, especially for the negative defect (Extended Data Fig. 4b,c). For the simulations, we did not include any experimental imperfections such as disorder and initial-state preparation fidelities. We further quantified the directional spreading of the defects by summing along the diagonals of the reference-subtracted occupations. When summing parallel to the equipotential lines, the occupation is only different from zero on the diagonals on which the defect atom and hole were initially placed. The growth by one additional diagonal for times *t*/*τ* > 0 can be explained by the above-mentioned processes, that is, the defect’s influence on the neighbouring atoms. As an additional characterization of the positive defect, we also studied the spreading on the zigzag-shaped equipotential line (Extended Data Fig. 5a, inset) instead of summing the reference-subtracted signal along the ROI diagonals. The result of this analysis is shown in Extended Data Fig. 5a. Here we again observe that the spread occurs along only one direction, as the immobile hole prevents the spread in the opposite direction. The latter is expected, as the hole can only move by second-order processes^8^. This is also evidenced by the density on the site of the hole remaining nearly unchanged.

Looking at this further, by comparing the increase of the densities on the sites initially occupied by the defect holes, we can also clearly observe the difference between the positive and the negative defects (Extended Data Fig. 5b). For the positive defect, the density increased only slightly, whereas for the negative defect we observed an immediate, fast increase, as here the hole is mobile in first order. Specifically, the hole of the negative defect can move in processes where neighbouring particles located on the equipotential line above the defect atom hop onto the defect atom and then to the site of the hole (Extended Data Fig. 4a, bottom right). The hole’s motion is restricted to its initial equipotential line. As for all other measurements on the spreading of defects on top of the chequerboard background, we attribute deviations from the theoretical expectations to disorder in the system and imperfect initial-state preparation, that is, the presence of additional, non-deterministic defects.

### Numerical methods for defects

The underlying physics in the Bose–Hubbard model is described by an effective Hamiltonian derived in ref. ^8^, which features HSF. In Extended Data Fig. 6, we compare the time evolution of the positive defect under this effective Hamiltonian with the time evolution of the original Bose–Hubbard model. We show the parity-projected on-site occupation and have subtracted a perfect chequerboard state (at *t*/*τ* = 0, that is, without time evolution) to better highlight the differences. It is noted that in Fig. 4 and Extended Data Figs. 4 and 5a, we instead subtract the theory calculations with a time-evolved version of the chequerboard for better comparison with the experimental data. In contrast to the effective model, the background chequerboard state is not completely frozen under time evolution with the Bose–Hubbard model. Nevertheless, this additional dynamics of the background does not strongly influence the dynamics of the mobile defect compared with the effective model. Therefore, we conclude that the underlying physics of the Bose–Hubbard model in the chosen limits are well captured by the effective model featuring HSF.

### Numerical methods for convergence

**Extended Data Fig. 7:**
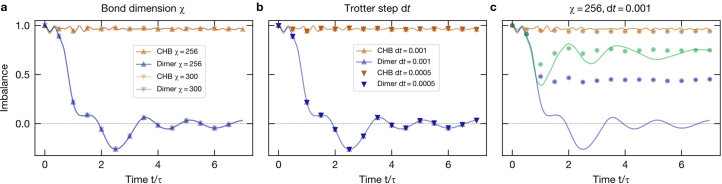
Convergence analysis and experimental versus “clean” time evolution. **a** Comparison of the time evolution for variable bond dimension *χ* for identical d*t* = 0.001. We find perfect agreement between *χ* = 256 and *χ* = 300, demonstrating the convergence of our code, assuming the experimental parameter regime *J* = 1, *U* = *Δ* = 18. **b** Similar analysis as in Extended Data Fig. 7a for *χ* = 256, yet with variable Trotter step size d*t*. We again observe excellent agreement. **c** Comparison of clean and imperfect system. Including experimental imperfections (circles) leads to a less pronounced decrease of the imbalance in comparison to the clean case (solid lines). Even with modified parameters, the effect of state-dependent dynamics is still clearly visible. Parameters used: *J*_*x*_ = 1, *J*_*y*_ = 1.1 × *J*_*x*_, *U* = *Δ*_*x*_ = *Δ*_*y*_ = 18 × *J*_*x*_, *δ* = [−2 × *J*_*x*_, 2 × *J*_*x*_], *N*_av,dimer_ ∈ [29, 100], *N*_av,squares_ ∈ [17, 100], *N*_av,CHB_ ∈ [10, 100], fidelity empty = 0.97, fidelity filled = 0.87. For all simulations, we have assumed a maximal filling of *N*_max_ = 2 per site. The on-site potential *δ* is sampled from a uniform distribution of the given width. Note that this is the same data as shown in Fig. 2a in the main text.

The data were obtained using tensor-network methods and exact diagonalization. All data were calculated by matrix-product-operator time evolution using the TeNPy package^46,47^, except for the time evolution with the effective model in Extended Data Fig. 6, which was performed with exact diagonalization. In Extended Data Fig. 7, convergence in the bond dimension and in the Trotter step is studied. In Extended Data Fig. 7a, the evolution of the imbalance for the chequerboard and dimer states shows perfect overlap for bond dimensions *χ* = 256 and *χ* = 300. For both curves, a Trotter step size of d*t* = 0.001 was used. In Extended Data Fig. 7b, the imbalance is compared for Trotter step sizes of d*t* = 0.001 and d*t* = 0.0005 at a fixed bond dimension of *χ* = 256.

### Numerical methods for imbalance

In Extended Data Fig. 7c, we compare the imbalance of the perfect case to the time evolution under imperfect conditions, similar to those of the experiment. For the latter, we have included deviations of all relevant quantities away from optimum, fidelities for state preparation and an additional random on-site potential (see the caption of Extended Data Fig. 7 for details). Each time step is averaged over *N*_av,dimer_ ∈ [29, 100], *N*_av,squares_ ∈ [17, 100], *N*_av,CHB_ ∈ [10, 100] different preparations. We find that the effect of state-dependent dynamics is still clearly visible also for experimental conditions. For the dimer state, the impact of experimental conditions is the strongest, which we attribute to the highest sensitivity to imperfect state preparation. In the case of the dimer state, all atoms have only one nearest neighbour. Removing this neighbour directly leads to a decrease in mobility and can induce frozen particles. By contrast, the squares state does not suffer from this effect on the same level. Each atom has three nearest neighbours, and therefore one missing neighbour does not lead to frozen sites.

## Online content

Any methods, additional references, Nature Portfolio reporting summaries, source data, extended data, supplementary information, acknowledgements, peer review information; details of author contributions and competing interests; and statements of data and code availability are available at 10.1038/s41586-024-08188-0.

## Data Availability

The data shown in the main text and Extended Data figures are available from the Edmond repository of the Max Planck Society at 10.17617/3.FPKWEQ.

## Code availability

The code underlying the numerical simulations is available from the Edmond repository of the Max Planck Society at 10.17617/3.FPKWEQ.
